# Evaluation and directed evolution for thermostability improvement of a GH 13 thermostable α-glucosidase from *Thermus thermophilus* TC11

**DOI:** 10.1186/s12896-015-0197-x

**Published:** 2015-10-21

**Authors:** Cheng Zhou, Yanfen Xue, Yanhe Ma

**Affiliations:** State Key Laboratory of Microbial Resources, Institute of Microbiology, Chinese Academy of Sciences, No. 1 West Beichen Road, Chaoyang District, Beijing, 100101 China; National Engineering Lab for Industrial Enzymes, Institute of Microbiology, Chinese Academy of Sciences, No. 1 West Beichen Road, Chaoyang District, Beijing, 100101 China

## Abstract

**Background:**

Thermal stable α-glucosidases with transglycosylation activity could be applied to the industrial production of oligosaccharides as well as conjugation of sugars to biologically useful materials. Therefore, α-glucosidases isolated from thermophiles have gained attention over the past decade. In this study, the characterization of a highly thermostable α-glucosidase and its thermostability improved mutant from newly isolated strain *Thermus thermophilus* TC11 were investigated.

**Results:**

The recombinant α-glucosidase (TtAG) from *Thermus thermophilus* TC11 was expressed in *Escherichia coli* BL21 (DE3) and purified. The purified enzyme had a molecular mass of 184 kDa and consisted of 59-kDa subunits; it showed hydrolytic activity for *p*NP-α-d-glucopyranoside (*p*NPG), sucrose, trehalose, panose, and isomaltooligosaccharides and very low activity for maltose. The highest specific activity of 288.96 U/mg was observed for *p*NPG at 90 °C and pH 5.0; Pb^2+^ provided a 20 % activity increase. TtAG was stable at 70 °C for more than 7 h and had a half-life of 195 min at 80 °C and 130 min at 90 °C. Transglycosylation activity was also observed with sucrose and trehalose as substrates. TtAG showed differences on substrate specificity, transglycosylation, multimerization, effects of metal ions and optimal pH from other reported *Thermus* α-glucosidases. One single-substitution TtAG mutant Q10Y with improved thermostability was also obtained from random mutagenesis library. The site-saturation mutagenesis and structural modelling analysis indicated that Q10Y substitution stabilized TtAG structure via additional hydrogen bonding and hydrophobic interactions.

**Conclusion:**

Our findings indicate that TtAG is a highly thermostable and more acidic α-glucosidase distinct from other reported *Thermus* α-glucosidases. And this work also provides new insights into the catalytic and thermal tolerance mechanisms of α-glucosidases, which may guide molecular engineering of α-glucosidase and other thermostable enzymes for industrial application.

**Electronic supplementary material:**

The online version of this article (doi:10.1186/s12896-015-0197-x) contains supplementary material, which is available to authorized users.

## Background

Glycoside hydrolases (GHs), catalyzing the hydrolysis of glycosidic linkages, are widely distributed in the natural world, and play essential roles in the carbohydrate metabolism [1]. In the CAZy database, GHs are classified into 133 families based on sequence similarity (http://www.cazy.org) [2]. Among GH families, GH family 13 is the largest family and contains various enzymes such as α-Amylases (EC 3.2.1.1), cyclodextrin glucanotransferases (EC 2.4.1.19), branching enzymes (2.4.1.18) and α-glucosidases (EC 3.2.1.20). GH family 13 enzymes show low similarity within their amino acid sequences, and are further divided into 40 subfamilies [3]. They also contain four short conserved regions (regions I–IV) including essential amino acid residues for catalysis [4]. GH family 13 contains several exo-glucosidases: α-glucosidase, oligo-1,6-glucosidase (EC 3.2.1.10, O16G) and dextran glucosidase (EC 3.2.1.70, DG). Most of these enzymes show high amino acid sequence similarity, and are classified into GH family 13 subfamily 31 (GH13_31) [5].

α-Glucosidases are typical exo-type amylolytic hydrolases that release α-glucose from non-reducing ends of oligosaccharides and polysaccharides [6] and commonly associate with other amylolytic enzymes, which completely degrade and utilize starch as a carbon source [7]. They are widely distributed among microorganisms, plants and animals, and take part in the glycogen metabolism of higher organisms and nutrient uptake and processing of bacteria [8]. α-Glucosidases are generally involved in the last step of starch degradation and are the second most important enzymes during the early stages of raw starch hydrolysis [9]. In addition to hydrolytic activity, some α-glucosidases possess transglycosylation activity that could be applied to the industrial production of oligosaccharides as well as conjugation of sugars to biologically useful materials [10–14]. Specifically, there is increased interest in applying the transglycosylation activity of α-glucosidases to the biosynthesis of bioactive compounds owing to the specificity, efficiency, and safety of the enzymatic reaction [15–18].

Numerous α-glucosidases have been characterized, with the majority from mesophilic organisms. Industrial application of these enzymes requires stability at high temperatures as well as toward common denaturant agents, and therefore, enzymes isolated from thermophiles have gained attention over the past decade [7]. There are many thermostable α-glucosidases from different thermophilic and hyperthermophilic microorganisms such as *Sulfolobus tokodaii* [7], *Geobacillus toebii* [19], *Thermus caldophilus* [20], *Thermoplasma acidophilum* [21], *Bacillus stearothermophilus* [22] and *Thermus thermophilus* [23, 24] have been discovered and characterized, and several mesophilic α-glucosidases have been engineered by mutagenesis to enhance enzyme thermostability [25, 26].

*Thermus thermophilus* is a thermophilic bacterium with optimal growth temperatures of approximately 70–75 °C and produce several enzymes of considerable biotechnological interest, including proteases, phosphatases, catalases, DNA processing enzymes, and α-glucosidases [27]. α-Glucosidases isolated from *T. thermophilus* HB8, *T. thermophilus* HB27, and *T. caldophilus* GK24 have been characterised with regard to their substrate specificity [20, 23, 24], which are different from that of the majority of known α-glucosidases. Whereas typical enzymes favour the α-1,4 glycosidic bonds of maltose or maltooligosaccharides [28], *Thermus* α-glucosidases preferentially hydrolyse the α-1,6 bonds in isomaltose, α-1,2 bonds in sucrose, or α-1,1 bonds in trehalose. In addition, they are thermostable and show transglycosylation activity with different substrates.

*Thermus thermophilus* TC11, isolated from a hot spring in Yunnan province of China, demonstrates high α-glucosidase activity even at 90 °C (data not published). Herein, the α-glucosidase (TtAG) gene from *T. thermophilus* TC11 was cloned and expressed in *Escherichia coli*. Some properties such as multimerization, effects of metal ions, optimal pH, substrate specificity and transglycosylation of recombinant TtAG were different from those of the previously described *Thermus* α-glucosidases, even though they have high sequence identities (>90 %). We also constructed and screened a random mutagenesis library and obtained one TtAG mutant with improved thermostability, which was analysed using site-directed mutagenesis and 3D structure modelling. Our findings further the understanding of molecular mechanisms underlying the thermostability of α-glucosidases and may facilitate the engineering of highly thermostable enzymes for industrial applications.

## Results

### Sequence analysis of TtAG

The α-glucosidase gene *ttag* (GenBank accession number: KP765743) cloned from *T. thermophilus* TC11 contains 1,587 bp and encodes a 528-amino-acid protein with a predicted molecular mass of 61.8 kDa. The deduced protein sequence showed the highest homology to microbial annotated α-glucosidases from *T. thermophilus* HB27 (GenBank No.WP_011172564), *T. thermophilus* HB8 (GenPept No.YP143747) and *T. caldophilus* GK24 (GenBank No. AF096282), oligo-1,6-glucosidases from *Bacillus flavocaldarius* (GenBank No.BAB18518), *Thermus.* sp. RL (GenBank No. EIA39407) and *Meiothermus silvanus* DSM 9946 (GenBank No.WP_013157279) with 96 %, 88 %, 87 %, 87 %, 87 %, and 70 % identity, respectively. Among these six α-glucosidases, the enzymes from *B. flavocaldarius*, *T.* sp. RL, and *M. silvanus* DSM 9946 had not yet been reported. The amino acid sequence alignment of TtAG with these five enzymes (Additional file 1: Figure S1) displayed that TtAG contains the four short conserved regions as region I 95-DLVPNH, region II 193-GFRVDVLWL, region III 244-EMRQ, and region IV 321-VLGNHD, indicating that TtAG belongs to glycoside hydrolase (GH) family 13 of clan GH-H [4].

### Purification and properties of the recombinant TtAG

The *ttag* gene was successfully expressed in *E. coli* BL21 (DE3), and the protein was purified to homogeneity by a two-step process, including heat treatment and His-Tag affinity chromatography. The final product showed an approximate 9.5-fold increase in purity with a recovery of 86.5 % activity relative to that of the crude enzyme (Table 1). The specific activity of the purified enzyme was 288.96 U/mg with *p*NPG as a substrate. Interestingly, the total TtAG activity did not decrease; rather, it increased by 11.9 % after heating at 70 °C for 30 min, when approximately 72 % of the *E. coli* total proteins were denatured.

**Table 1:** Purification of recombinant TtAG

Preparation	Total protein (mg)	Total activity (U)	Yield (%)	Specific activity (U/mg)	Purification fold
Supernatant of crude extract	89.4	2772.2	100.0	31.01	1.0
Supernatant after heating treatment	25.5	3312.0	119.0	129.88	4.2
Affinity (His-Tag column)	8.3	2398.4	86.5	288.96	9.3

**Fig. 1:**
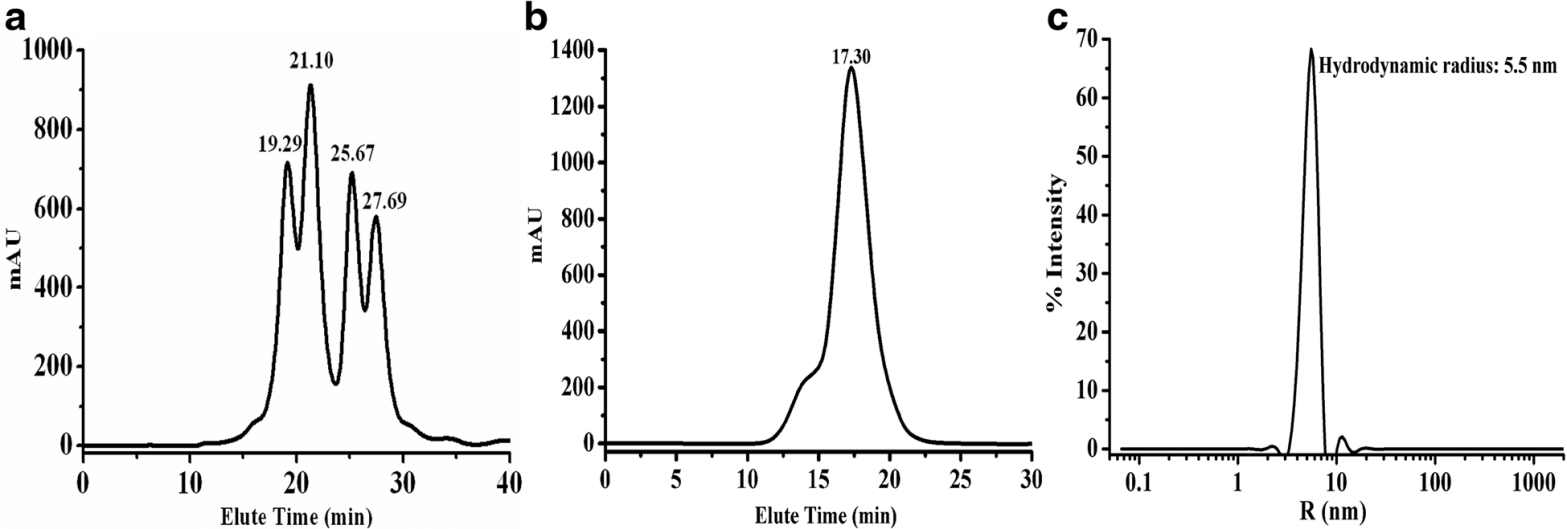
The gel filtration and dynamic light scattering (DLC) of the native recombinant TtAG. **a** The gel filtration of molecular standard marker. **b** The gel filtration of the native recombinant TtAG. **c** The regularization graph of the native recombinant TtAG in DLC experiment. The Rayleigh Spheres model was used. The x-axis indicates the hydrodynamic radius

The purified recombinant TtAG was homogeneous with an approximate molecular mass of 60 kDa in SDS-PAGE (Additional file 1: Figure S2), which is consistent with the theoretical mass, based on the amino acid sequence. However, gel filtration chromatography showed that the molecular mass of the native TtAG was beyond the detection range of 150 kDa, indicating that native TtAG is an oligomer (Fig. 1a and b). To accurately determine the molecular mass of native TtAG, we performed dynamic light scattering (DLS) analysis, which detected a peak with a hydrodynamic radius of 5.5 nm (Fig. 1c), corresponding to a molecular mass of 184 kDa, suggesting that native TtAG exists in solution as a trimer, whereas the α-glucosidase AglH_HB27_ from *T. thermophilus* HB27 is a dimer [23].

A previous study on the catalytic mechanism of α-glucosidase from *Geobacillus* sp. strain HTA-462 (GSJ), which shows the highest sequence similarity with TtAG in Protein Data Bank (PDB) data base and also belongs to GH family 13, identified conserved amino acids D199, E256, and D326 as the catalytic residues and H103 and H325 as the substrate binding sites [8]. Sequence alignment demonstrated that D197, E264, D326, H100, and H325 of TtAG were highly corresponded and conserved to these sites of GSJ, respectively. Three TtAG mutants, carrying D197N, D326N and E264Q substitutions, were obtained by site-directed mutagenesis to experimentally confirm the catalytic residues, and all the three mutants were inactive. The CD spectra were also checked and the gross conformation of the three mutants did not change (data not shown). So D197, D326, and E264 were considered as TtAG catalytic sites and H100 and H325 as its substrate-binding sites.

### Physical properties of the recombinant TtAG

The effect of pH and temperature on recombinant TtAG catalytic activity was determined using *p*NPG as a substrate. TtAG showed maximum activity at pH 5.0 and 90 °C and retained more than 60 % and 50 % activity at pH 4.5-8.0 and temperatures of 70 °C–95 °C, respectively. Importantly, it retained 35 % activity at 100 °C, but showed almost no activity below 50 °C. TtAG was stable over a wide range of pH values (4–9) at 50 °C, retaining over 80 % of its original activity after a 30-min incubation. TtAG also demonstrated significant thermostability; it remained fully active after a 7-h incubation at 70 °C and showed a half-life of 210 min at 80 °C and 130 min at 90 °C, suggesting that it could be a good candidate for application in industrial settings. However, the thermostability of TtAG was negatively affected by NaCl indicating that salt was an adverse factor for enzyme stability at high temperatures.

The effect of various metal ions and chemicals on TtAG enzymatic activity was examined after a 30-min incubation at 50 °C and pH 6.0. Al^3+^, Hg^2+^, Fe^3+^, Li^+^, Ce^4+^, Ag^+^, Fe^2+^, Ni^2+^, and Cu^2+^ significantly inhibited TtAG activity, whereas Mg^2+^, Mn^2+^, Ca^2+^, and EDTA had no effect, and Pb^2+^ produced a 20 % increase. TtAG was tolerant to 8 M urea, retaining approximately 95 % activity, but was sensitive to guanidine hydrochloride and SDS, demonstrating only 20 % and 30 % activity after incubation with 4 M guanidine hydrochloride and 1 % SDS, respectively.

### Substrate specificity and Transglycosylation of TtAG

The hydrolytic activity of TtAG with various substrates was examined at 90 °C; kinetic parameters are shown in Tables 2. TtAG was capable of cleaving sucrose (α-1,2 bond), trehalose (α-1,1 bond), panose (α-1,6 and α-1,4 bonds), isomaltose (α-1,6 bond), isomaltotriose, and aryl-substrate *p*NPG, with the highest specific activity of 288.96 U/mg for *p*NPG (Table 2). No activity was detected for *p*-nitrophenyl-β-d-glucopyranoside, raffinose, melibiose, melezitose, maltooligosaccharides, starch, amylopectin, glycogen, pullulan, or dextrin. Very low activity (0.5 U/mg) for maltose (α-1,4 bond) was observed only when the reaction time was increased to 2 h and excess enzyme was added. The *Km* for *p*NPG was 0.48 mM, which was lower than that for the other substrates. The TtAG substrate preference was as follows: *p*NPG > isomaltotriose > isomaltose > trehalose > panose > sucrose (*k*_*cat*_/*Km* values of 1372.08, 12.15, 9.39, 7.02, 4.87, and 2.46 s^−1^ · mM^−1^, respectively; Table 2). Among natural substrates, TtAG showed higher specificity for isomaltooligosaccharide than for other oligosaccharides. Overall, these results indicate that TtAG acts as an oligo-1,6-glucosidase, which is consistent with the characteristics of GH13 α-glucosidases. α-glucosidases are further classified into three types according to their substrate specificity. Type I hydrolyses aryl glucosides such as *p*NPG faster than short malto-oligosaccharides. However, Type II is more active on maltose and has low activity towards aryl glucosides, whereas Type III resembles Type II, but hydrolyses oligosaccharides and starch at similar rates [29]. From this substrate specificity, TtAG belongs to Type I α-glucosidase.

**Table 2:** Kinetic parameters of recombinant TtAG for hydrolysis of various substrates

Substrate	*K*_*m*_ (mM)	*K*_*cat*_ (s^−1^)	*K*_*cat*_/*K*_*m*_ (s^−1^ mM^−1^)
*p*NPG	0.48	658.60	1372.08
Sucrose	30.53	74.64	2.46
Trehalose	19.96	140.04	7.02
Panose	18.57	90.42	4.87
Isomaltose	10.59	99.51	9.39
Isomaltotriose	15.58	189.3	12.15
Maltose	n d.	n d.	n d.

**Fig. 2:**
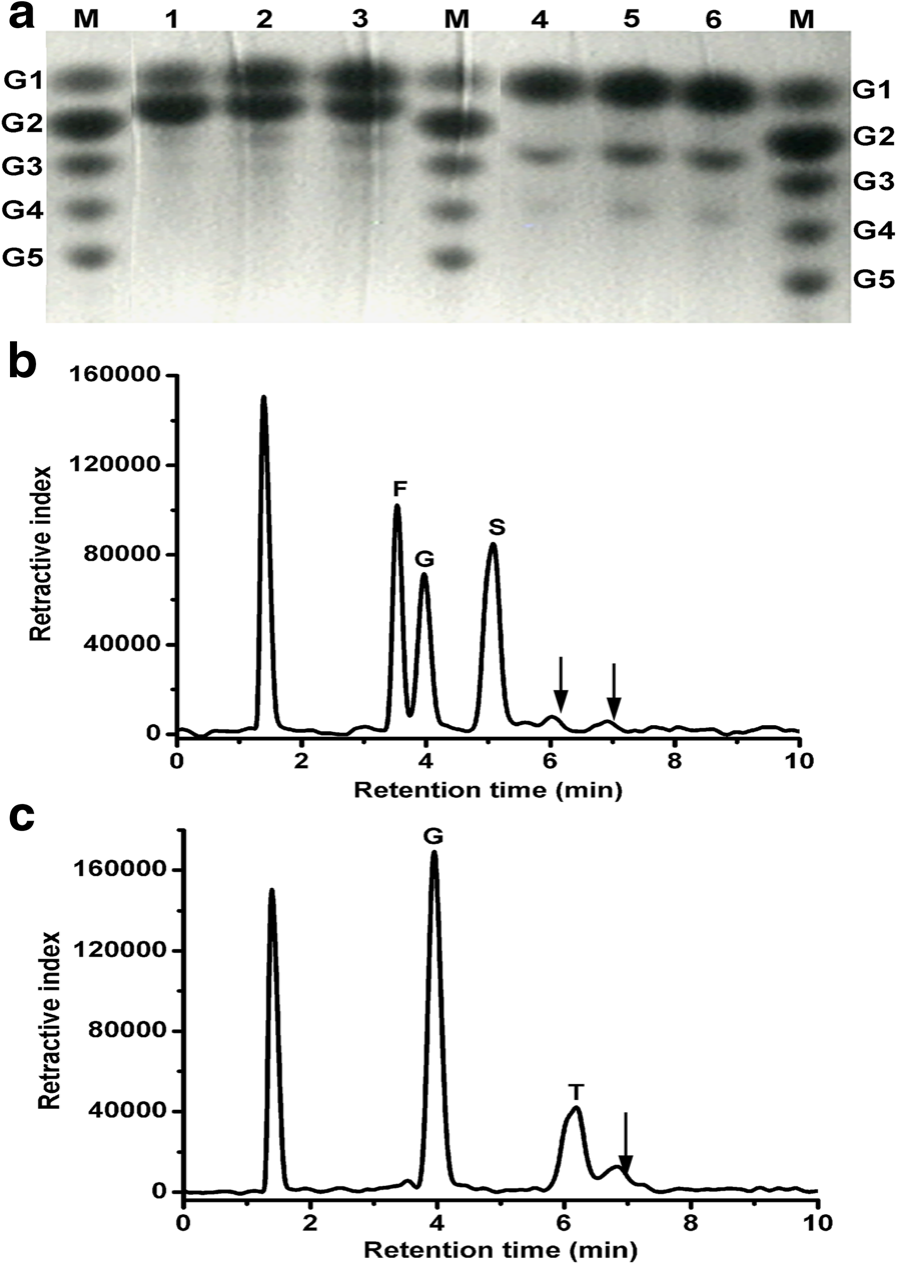
TLC and HPLC chromatogram analysis of transglycosylation products. **a** TLC analysis of transglycosylation products when sucrose and trehalose at 300 mM were used as substrates for different incubation time. Lane 1, sucrose for 3 h; lane 2, sucrose for 10 h; lane 3, sucrose for 20 h; lane 4, trehalose for 3 h; lane 5, trehalose for 10 h; lane 6, trehalose for 20 h; lane M, marker. G1, glucose; G2, maltose; G3, maltotriose; G4, maltotetraose; G5, maltopentaose. **b** and (**c**) HPLC chromatogram of the reaction mixture after 20 h of incubation when sucrose or trehalose was used as the substrate. F, fructose; G, glucose; S, sucrose; T, trehalose. The arrows indicate the new transglycosylation products

To determine whether TtAG could perform transglycosylation, it was incubated with 300 mM isomaltotriose, isomaltose, trehalose, panose, and sucrose for 3 h. TLC analysis of the reaction products revealed transglycosylation activity only with sucrose or trehalose as substrates. Further analysis of TtAG transglycosylation activity with trehalose and sucrose showed that the yield of transglycosylation products increased with the incubation time (up to 20 h; Fig. 2a). The transglycosylation activity of TtAG was confirmed by detection of reaction products after TtAG incubation with 300 mM sucrose or trehalose for 20 h. HPLC analysis revealed three major peaks corresponding to sucrose, glucose, and fructose, and two minor peaks corresponding to the products of a transglycosylation reaction with sucrose (Fig. 2b); similarly, a small peak indicating a transglycosylation product was detected after TtAG reaction with trehalose (Fig. 2c). These results further confirmed the transglycosylation activity of TtAG with sucrose and trehalose.

### Screening and biochemical properties of TtAG mutants with improved thermostability

Random mutagenesis by error prone PCR (ep-PCR) was used to get thermostability improved variant of TtAG. The ep-PCR library constructed using the MEGAWHOP method [30] contained approximately 30 % inactive TtAG variants. In general, one to five nucleotide mutations per gene were observed. The library was expressed using the auto induction system; the standard deviation was 12.8 %, which conforms to the 10 % standard deviation in screening systems successfully used in directed evolution experiments [31, 32]. The activity ratio of heat-treated to untreated bacteria was designated as residual activity and used to select mutants with improved thermostability; the selection criterion was an increase of at least 10 % in the residual activity of a mutant compared to the wild type after heat treatment. Approximately 2,700 ep-PCR library clones were screened and one mutant with increased thermostability carrying a single amino acid substitution of Q10Y was selected. Under the screening conditions, the residual activity ratio of the wild-type TtAG was approximately 15 %, whereas that of Q10Y mutant was approximately 38 %.

The Q10Y mutant and wild-type TtAG were expressed in *E. coli* BL21 (DE3) and their thermostability was determined based on T_50_^15^ and ΔT_50_^15^ values. The T_50_^15^ of Q10Y was 97 °C, with ΔT_50_^15^ of 4 °C compared to the wild-type TtAG (T_50_^15^ of 93 °C). These results indicate that the thermostability of the Q10Y mutant was significantly increased compared to that of the wild-type enzyme. The substrate specificity of Q10Y mutant was also determined as similar to the wild-type TtAG (data not shown). The hydrolytic activity and kinetic parameters of the mutated and wild-type enzymes were determined at the optimal reaction conditions. The specific activity of Q10Y with *p*NPG was 236.9 U/mg which decreased to approximately 82 % of that of wild-type TthAG, indicating that the increase in thermostability was accompanied by a loss of enzymatic activity. The *Km* of Q10Y was 0.46 mM and similar to that of the wild-type indicating similar substrate binding ability. However, the *k*_*cat*_ of Q10Y (356.57 s^−1^) decreased 46 % which resulted in about 44 % decrease of the *k*_*cat*_/*Km* ratio (775.15 s^−1^ mM^−1^). These data indicate that the mutation of Q10Y affected not only the thermostability but also the catalytic activity of TtAG, suggesting that the mutated residue are located in close proximity to the active and/or substrate-binding sites and can affect catalysis. In order to further confirm the influence of Q10 site for thermostability, saturation mutagenesis of the Q10 site was done by site-directed mutagenesis. The results demonstrated that the T_50_^15^ values of all 18 other mutants carrying single amino acid substitutions decreased compared to that of the wild-type enzyme (data not shown), which means that only the Q10Y substitution in this site improved the thermostability of TtAG. This single substitution Q10Y was firstly reported responsible for improvement of the thermal tolerance of α-glucosidase, and may help for guiding molecular engineering of other α-glucosidases for thermostability improvement.

## Discussion

In this study, we successfully expressed, purified and characterized the thermostable α-glucosidase TtAG from *T. thermophilus* TC11. Although TtAG and other reported *Thermus* α-glucosidases similarly cannot hydrolyse the disaccharides cellobiose and melibiose or the trisaccharides raffinose and melizitose and are inactive for polysaccharides such as starch, amylopectin, glycogen, pullulan, and dextrin, there are still many differences in biochemical properties and substrate specificity between TtAG and other *Thermus* α-glucosidases, even though they demonstrate more than 90 % similarity with respect to amino acid sequence. Ca^2+^ can activate the α-glucosidases AglH_HB27_ and AglH_HB8_ from *T. thermophilus* HB8 and TcaAG from *T. thermophilus* GK24, whereas Pb^2+^ inhibits their activity. In contrast, TtAG is activated by Pb^2+^, whereas Ca^2+^ has no effect. Pb^2+^, as a heavy metal ion, generally inhibits the activity of most enzymes, including α-glucosidases [14, 24]. Therefore, TtAG activation by Pb^2+^ may indicate an interesting property different from that of other α-glucosidases. However, about this experimental observation, we actually cannot understand why based on our current knowledge. The optimal pH for TtAG activity is 5.0, whereas that for AglH_HB8_, AglH_HB27,_ and TcaAG was higher (5.8, 6.2, and 6.5, respectively) [20, 23, 24], suggesting increased acid resistance. Although TtAG is similar to the other three *Thermus* α-glucosidases in terms of optimal temperature (90 °C) and pH tolerance, it demonstrates significant variations in other properties, indicating that TtAG is an α-glucosidase with distinct biochemical characteristics.

The substrate specificity and catalytic efficiency of TtAG and other reported *Thermus* α-glucosidases are summarized in Table 3. Similar to AglH_HB27_, TtAG can hydrolyse trehalose (α-1,1-glucosidic linkage). However, the activity of AglH_HB27_ for isomaltose (α-1,6), isomaltotriose, and sucrose (α-1,2) was only 63.8 %, 30.5 %, and 8.0 % of that for trehalose [23], while the activity of TtAG for the same substrates was 96.5 %, 181.6 % and 46.1 %, respectively, indicating a preference of TtAG for isomaltotriose over trehalose, which is different from the substrate specificity of AglH_HB27_. In contrast to TtAG and AglH_HB27_, the α-glucosidase AglH_HB8_ and TcaAG cannot hydrolyse trehalose [20, 24] and prefer isomaltose, whereas TtAG and AglH_HB27_ are most active with *p*NPG and trehalose, respectively. Meanwhile, all these four enzymes belong to Type I α-glucosidases according to their substrate specifities. The activity towards sucrose may made TtAG has potential application in high fructose syrup production.

**Table 3:** Substrate specificity for oligosaccharides of α-glucosidase TtAG, AglH_HB27_, AglH_HB8_ and TcaAG from *Thermus* strains

Substrate	Glycosidic linkage	Relative activity (%)			
		TtAG	AglH_HB27_	AglH_HB8_	TcaAG
Sucrose	Glu-α-1,2-Fru	47.8 (46.1)	12.5 (8.0)	8.4	59.3
Trehalose	Glu-α-1,1-Glu	103.7 (100.0)	156.7 (100.0)	0.0	0.0
Isomaltose	Glu-α-1,6-Glu	100.0 (96.5)	100.0 (63.8)	100.0	100.0
Cellobiose	Glu-β-1,4-Glu	0.0	0.0	0.0	0.0
Melibiose	Gla-α-1,6-Glu	0.0	0.0	0.0	0.0
Maltose	Glu-α-1,4-Glu	3.7 (3.5)	15.1 (9.7)	2.8	1.6
Melizitose	Glu-[α-1,3]-Fru-β-2,1-Glu	0.0	0.0	0.0	0.0
Isomaltotriose	Glu-[α-1,6]-Glu-α-1,6-Glu	188.2 (181.6)	47.8 (30.5)	45.2	4.8
Raffinose	Gla-[α-1,6]-Glu-α-1,2-Glu	0.0	0.0	0.0	0.0
Panose	Glu-[α-1,6]-Glu-α-1,4-Glu	62.5 (60.3)	56.1 (35.8)	42.1	44.6

The transglycosylation activity reported for many α-glucosidases has been exploited in biotechnology to produce food oligosaccharides or to conjugate sugars with biologically active materials [14]. AglH_HB27_ catalyses transglycosylation only in the presence of higher levels of trehalose or isomaltose and incubation for up to 24 h, whereas TcaAG shows transglycosylation activity only with sucrose or isomaltose; however, TtAG demonstrated transglycosylation with sucrose and trehalose after only 3 h, suggesting higher transglycosylation activity compared to that of other *Thermus* α-glucosidases. Although TtAG shares approximately 90 % amino acid sequence identity with the homologous enzymes from *T. thermophilus* strains HB27 and HB8 and *T. caldophilus* GK24, it has different substrate specificity, catalytic efficiency (Table 3), and transglycosylation activity. It has been suggested that enzymes with high amino acid identity but different substrate specificity may have evolved through a limited number of amino acid substitutions to give rise to enzymes with broader substrate specificity or other related activities [33].

**Fig. 3:**
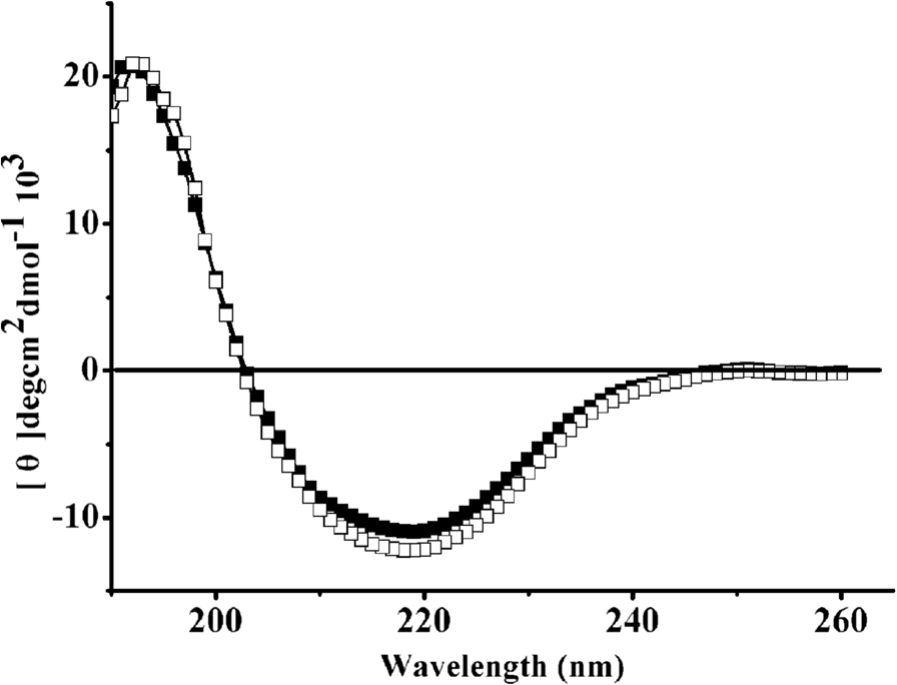
Far-UV CD spectra of TtGA and mutant Q10Y. (□) TtAG; (■) Q10Y

**Fig. 4:**
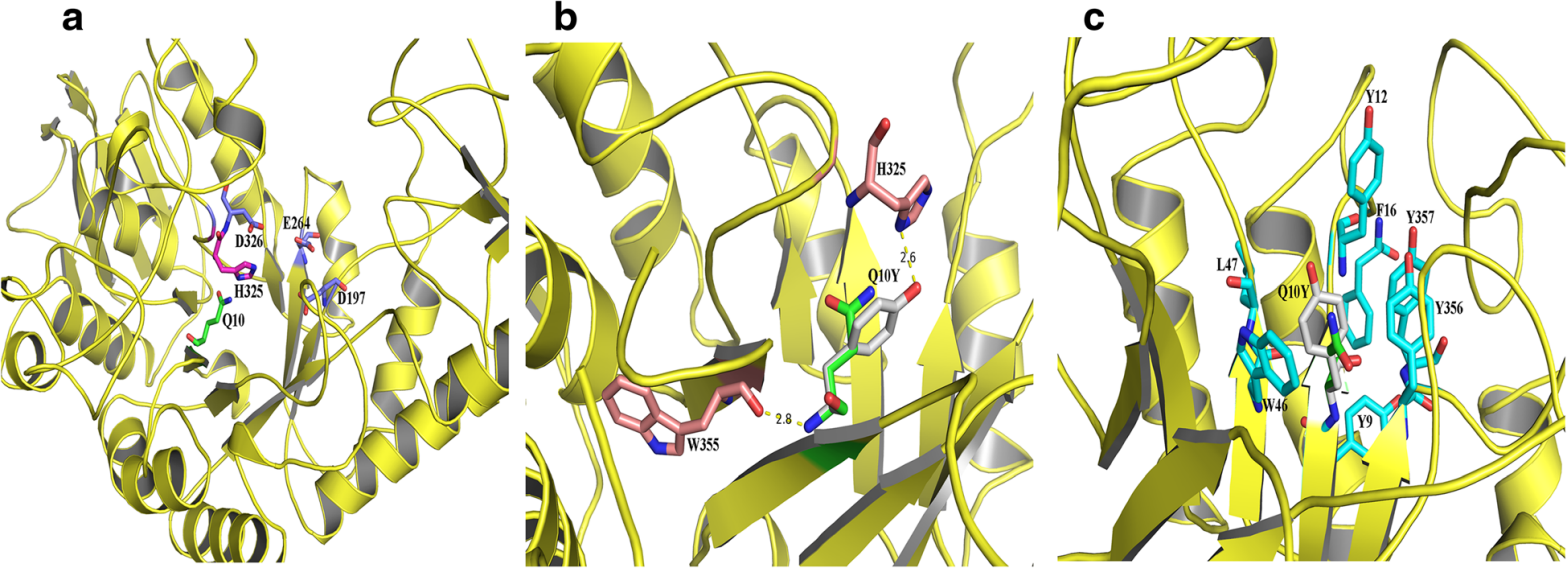
Location and interaction of Q10 site in the modelled 3D structure of TtAG. **a** Location of the Q10 site. **b** Hydrogen bond interaction of the Q10 site. **c** Hydrophobic interaction of the Q10 site. The structure was modelled by SWISS-MODEL online and the figure was developed using Pymol™ 1.3.X

The CD spectra were analyzed to determine whether the mutation affected the secondary structure. The results showed that there were no distinct changes between wild-type TtAG and the Q10Y mutants (Fig. 3). This meant that the thermostability improvements were not caused by secondary structural change. The TtAG mutant Q10Y demonstrated increased thermostability but decreased enzymatic activity. To determine how the Q10Y mutation affected TtAG thermostability, the 3D structure of TtAG was modelled based on the structure of α-glucosidase GSJ from *Geobacillus* sp. strain HTA-462 (PDB:2ze0) as a template, using the SWISS-MODEL software online. The PROCHECK analysis showed that 90.9 % of the residues were in the most favoured regions and 8.7 % of the residues were in additional regions, indicating the reliability of the modelled structure. As shown in Fig. 4a, Q10 is located in the β-barrel of the catalytic domain, in close proximity to the catalytic and substrate-binding sites D326 and H325, respectively; therefore, Q10Y substitution would probably influence TtAG substrate binding and/or catalytic efficiency, resulting in a decrease in enzymatic activity. Q10Y substitution in the TtAG catalytic centre enhances TtAG thermostability, probably by stabilisation of the catalytic domain. In the structure of the homologous enzyme GSJ, a hydrogen bond is formed between Q13 and I361, located on different β-sheets [8]. In TtAG, the homologous amino acids Q10 and W355, respectively, are also located on two adjacent β-sheets with the distance of 2.8 Å (Fig. 4b), which is sufficient for the formation of a hydrogen bond. Similarly, hydrogen bonds can be formed between the amino group of Y10 and the carboxyl of W355 and between the hydroxyl of Y10 benzene ring and the amino group of His325, which are also located in the adjacent β-sheets with a distance of 2.6 Å (Fig. 4b). Saturation mutagenesis of the Q10 site demonstrated that the T_50_^15^ values of all 18 other mutants carrying single amino acid substitutions (except Q10Y) decreased compared to that of the wild-type enzyme, likely because the mutated proteins could not form additional hydrogen bonds. Because hydrogen bonding is considered to directly influence protein thermostability [34], we hypothesize that additional hydrogen bonding in the Q10Y mutant could account for its increased thermostability. In addition, among the other 18 saturation mutants, only Q10E demonstrated the same enzymatic activity as the wild type TtAG (data not shown), probably because of similarities in terms of structure and size between glutamine and glutamiс acid, which differ only in charge (neutral for Q and negative for E), suggesting an important role of Q10 for TtAG enzymatic activity.

Q10 is located also close to a possible hydrophobic core formed by Y9, Y12, F16, W46, L47, Y356, and Y357 (Fig. 4c). A previous study determined that the hydrophobic amino acid content is marginally higher in thermophiles than in mesophiles, which can increase protein rigidity and hydrophobicity [35], thus suggesting a role of hydrophobic interactions in protein thermostability [36–38]. Tyrosine is more hydrophobic than glutamine; hence, Q10Y substitution may strengthen hydrophobic interactions between this and other tyrosines of the TtAG hydrophobic core, resulting in enhanced thermostability. However, even though some other amino acids are more hydrophobic than glutamine, only the substitution of Q10Y stabilized TtAG; therefore, we suggest that a combination of hydrogen bonding and hydrophobic interactions of mutation Q10Y resulted in improved thermostability for TtAG.

## Conclusions

In summary, a highly thermostable and acidic α-glucosidase, TtAG, from *T. thermophilus* TC11 was cloned, expressed, and characterized. Although TtAG shares approximately 90 % amino acid identity with the homologous α-glucosidases from other *T. thermophilus* strains, it has distinct biochemical characteristics, which indicates that TtAG is an α-glucosidase with potential biotechnological applications. The TtAG mutant carrying the single amino acid substitutions Q10Y demonstrated enhanced thermostability. Structure modelling and site-directed mutagenesis demonstrated that hydrogen bonding and hydrophobic interactions were responsible for the increased thermostability imparted by Q10Y. Our findings provide new insights into the catalytic and thermal tolerance mechanisms of α-glucosidases, which may guide molecular engineering of thermostable enzymes for industrial applications. Furthermore, crystallization and real 3D structure resolution of TtAG in the future would deepen the understanding of catalysis by α-glucosidase and facilitate the thermal adaptation of thermostable enzymes.

## Materials and methods

### Strains, plasmids, and media

*Thermus thermophilus* TC11, isolated from the hot spring of Tengchong in the Yunnan province of China, was obtained from the China General Microbiological Culture Collection Center (CGMCC 1.15099) and used as DNA source for cloning of the α-glucosidase (TtAG) gene. pET28a and *Escherichia coli* BL21 (DE3) (Novagen, USA) were used for gene expression. All of the restriction enzymes used in molecular procedures were from TaKaRa Biotechnology (Dalian, China). D*pn*I was from New England Biolabs (Ipswich, USA). The oligosaccharides were from Sigma-Aldrich (St. Louis, USA). The isopropyl-β-D-thiogalactopyranoside (IPTG), kanamycin, imidazole and protein denaturants were from Merck (Darmstadt, Germany). All the other chemicals used were of reagent grade.

### Construction of expression plasmid and transformation of cells

The TtAG gene (*ttag*) was amplified from the genomic DNA of *Thermus thermophilus* TC11 by PCR with a primer pair of the forward (5′-GCTAGCTAGCATGCTTCAAAGAAC-3′, where the underline indicates the NheI site) and the reverse (5′-CCGGAATTCCTATTTCACTACAATC-3′, the underline indicates the EcoRI site) and pfu DNA polymerase (Takara). The PCR product was purified using the Gel Extraction Kit (OMEGA Bio-tek, USA) and then digested with NheI and EcoRI to insert the digested pET28a vector. The resultant recombinant plasmid, pET28a-*ttag*, was transformed into *E. coli* BL21 (DE3) for gene expression.

### Gene expression and protein purification

*E. coli* BL21 (DE3) harbouring the recombinant plasmid was cultured in 0.5 L of LB medium containing kanamycin (60 μg/ml) until the OD_600_ reached 0.6. IPTG was added at a final concentration of 1 mM, and the cells were continuously cultivated at 37 °C for 5 h. Cells were harvested by centrifugation at 6,000 × g at 4 °C for 15 min, washed with binding buffer (20 mM Tris–HCl buffer containing 500 mM NaCl and 5 mM imidazole, pH 7.9) and then suspended in 50 ml of the same buffer. The suspended cells were disrupted by sonication and the supernatant was obtained by centrifugation at 12,000 × g for 20 min at 4 °C. The supernatant was incubated at 70 °C for 30 min and then centrifuged at 12,000 × g for 30 min at 4 °C. The second supernatant was loaded onto a His•Bind column (5PKG) (Novogen, Germany). The column was washed with 10 ml of binding buffer and subsequently with 15 ml of washing buffer (20 mM Tris–HCl buffer containing 500 mM NaCl and 60 mM imidazole, pH 7.9). Finally, the protein was eluted with 3 ml of elution buffer (20 mM Tris–HCl buffer containing 500 mM NaCl and 1 M imidazole, pH 7.9). The obtained protein solution was desalted using a desalting column (GE Healthcare, England) with 20 mM Tris–HCl buffer (pH 7.5). The protein concentration was determined using the Bradford method [39] with bovine serum albumin as a standard. The purity of the protein was examined by SDS-PAGE and stained by Coomassie Brilliant Blue.

### Molecular mass determination and circular dichroism spectra

The apparent molecular mass of the recombinant enzyme was determined by both gel filtration chromatography Superdex 10/300 colum using cytochrome c (12.4 kDa), carbonic anhydrase (29 kDa), bovine serum albumin (66 kDa) and alcohol dehydrogenase (150 kDa) (Sigma, St. Louis, U.S.A.) as molecular mass standard. The DynaPro dynamic light scattering (DLC) systems (Wyatt Technology, USA) were also used to determine the molecular mass and the multimerization state of the native recombinant TtAG. 0.1 mg/ml of TtAG in 20 mM Tris–HCl buffer (pH 7.5) was used and the DLC experiment was performed at 25 °C. The accurate molecular mass was obtained by hydrodynamic radium of the peak resulted by native recombinant TtAG. The monomer molecular mass was calculated from the putative amino acid sequence and estimated by SDS-PAGE.

Circular dichroism (CD) spectra of pure recombinant TtAG and the improved mutants were measured using the Jasco J-810 spectropolarimeter (Jasco, Japan) over a wavelength ranging from 190 to 260 nm under constant nitrogen flush. The bandwidth was set to 1 nm. The secondary structure content of the enzyme was estimated using the program JASCOW32. All spectra were recorded at room temperature, and three scans were averaged and blank-subtracted to give the spectra.

### Effect of pH, temperature and chemicals on enzyme activity and stability

The optimal pH was assayed at 90 °C in 0.1 M citric acid-0.2 M sodium phosphate buffer (pH 4.0-8.0) with 0.5 mM *p*-Nitrophenyl-α-D-glucopyranoside (*p*NPG). The effect of pH on enzyme stability was analyzed with enzyme being incubated in buffer from pH 3–11 at 50 °C for 30 min. The optimal temperature was assayed at 50-98 °C for 10 minutes with standard reaction buffer (citric acid (0.1 M)-sodium phosphate (0.2 M) buffer, pH 5.0). Thermal stability was analyzed by assessing enzyme activity after incubation at 70 °C, 80 °C and 90 °C for continuous time. To determine the effects of chemicals, the enzyme (0.1 mg/ml) was incubated with various metal ions and EDTA of 5 mM, and protein denatures of different concentration at 50 °C for 30 min. The residual activity was measured under the standard hydrolytic assay condition.

### Standard hydrolytic activity assays

α-Glucosidase hydrolytic activity was determined by measuring the release of *p*-nitrophenol from *p*NPG at 90 °C for 10 min in standard reaction buffer. The reaction was terminated by addition of equal volume of 1 M Na_2_CO_3_ solution. The absorbance of the liberated *p*-nitrophenol was measured at 410 nm. One unit of activity was defined as the amount of enzyme liberating 1 μmol of *p*-nitrophenol in one minute.

The activity on oligosaccharides and polysaccharides was determined by measuring the release of glucose at 90 °C for 15 min with 0.5 % (w/v) substrates in standard reaction buffer. Reactions were terminated by incubation for 10 min in ice water bath. Glucose was assayed with the glucose oxidase reagent from a glucose assay kit (Sigma Diagnostics no. 510). One unit of activity was defined as the amount of enzyme liberating 1 μmol of glucose in one minute.

### Transglycosylation activity analysis

The transglycosylation activity was determined using 5 μg of purified enzyme and 300 mM maltose, sucrose, or trehalose in the standard reaction buffer at 75 °C. After different time intervals, the reaction was terminated in a boiling water bath for 10 min. After centrifugation (10,000 × g, 10 min), 1-μl samples were spotted on Silica Gel 60 plates (Merck, Germany) and thin-layer chromatography (TLC) analysis was performed as described previously by Kanda et al. [40]. Transglycosylation product analysis was conducted by HPLC using a 4.6 mm ID × 150 mm Zorbax Carbohydrate Analysis column (Agilent Technology, USA) with acetonitrile/water (75/25, v/v) as the mobile phase at 2 ml/min and a refractive index detector. The column temperature was kept constant at 30 °C.

### Random mutagenesis library construction and screening

Random mutagenesis was performed by the standard error-prone PCR (ep-PCR) method with primers pairs (forward, 5′-ATGCTTCAAAGAAC-3′; reverse, 5′-CTATTTCACTACAATC-3′) and the plasmid pET28a-*ttag* as the template. The PCR amplification reactions were conducted with rTaq DNA polymerase (Takara) and Mn^2+^ added at 0.4 mM. The PCR products were purified using a Gel Extraction Kit (OMEGA Bio-tek, USA). The purified ep-PCR products were cloned into the expression plasmid pET28a by Phusion Hot Start DNA polymerase (Finzyme) following the Megaprimer PCR of Whole Plasmid (MEGAWHOP) method [30] with plasmid pET28a-*ttag* as a template. Following PCR, D*pn*I was added and the mixture was incubated overnight at 37 °C. The MEGAWHOP products were then transformed into *E. coli* BL21 (DE3) for expression and screening.

Colonies grown on LB-kanamycin (60 μg/ml) agar plates were transferred, by using toothpicks, into 96-well microtiter plates containing 150 μl of MDG non-inducing medium [41] in each well supplemented with kanamycin (60 μg/ml). Two wells of each 96-well microtiter plate contained wild-type TtAG colonies. After 20 h of cultivation in a microtiter plate shaker (Multitron II, Infors GmbH, 37 °C, 900 rpm, 70 % humidity), each well was replicated by using a replicator (EnzyScreen BV, Leiden, Netherlands) into a second series of 96-well microtiter plates containing 150 μl of induction medium MD-5052 [41] supplemented with kanamycin (60 μg/ml). The first set of plates was stored at −80 °C after addition of glycerol. The clones in the second set of plates were cultivated for 15 h in the microtiter plate shaker and used for screening.

A 20-fold dilution of the cell culture (30 μl) was transferred into a 96-well PCR plate and then incubated at 99 °C for 60 min in a thermal cycler (Mastercyler gradient; Eppendorf); then, 5 μl of the incubated cell culture was transferred into another 96-well PCR plate with 70 μl of Na_2_HPO_4_-citric acid buffer including 0.5 mM *p*NPG (pH 5.5) in each well. The reaction was performed at 90 °C for 5 min and then terminated by addition of 75 μl of 1 M Na_2_CO_3_ solution. The residual activity was determined by the absorbance at 410 nm. Mutants in which the residual activity (%) was at least 10 % higher than that of the wild-type enzyme were preliminarily considered to have improved thermostability.

### Thermostability determination of wild-type and mutant enzymes

The mutant enzymes with potential improvement of thermostability were expressed and purified as described above. T_50_^15^ and ΔT_50_^15^ (T_50_^15^ was the temperature at which the enzyme retained 50 % of the initial activity, and ΔT_50_^15^ was the difference between T_50_^15^ of the mutant and wild-type enzymes) were used to evaluate the thermostability of wild-type and mutant α-glucosidases according to the method of Eijsink et al. [42]. The purified recombinant wild-type enzyme (TtAG) and other mutated enzymes were incubated for 15 min at 90 °C to 99 °C at a concentration of 2 μg/ml in 20 mM sodium phosphate buffer (pH 7.5). The residual activity of the heated solution was assessed as described above.

### 3D structure modeling and site mutagenesis

The tertiary structure of TtAG was modeled by SWISS-MODEL server on line (http://swissmodel.expasy.org/), using the structure of α-glucosidase GSJ from *Geobacillus* sp. strain HTA-462 (PDB: 2ze0) as a template, which showed the highest identity with TtAG in the PDB database and also belongs to GH family 13. Figures were developed using Pymol™ 1.3.X. The reliability of the modelled structure of TtAG was checked by PROCHECK software online (http://www.ebi.ac.uk/thornton-srv/software/PROCHECK/). Site mutagenesis was done using the QuikChange® Site-Directed Mutagenesis Kit (Stratagene, USA). The recombinant plasmid, pET28a-*ttag*, was used as the template. The mutagenesis PCR products were transformed into *E. coli* XL1-Blue (Novagen) directly after D*pn*I digestion. DNA sequencing was performed by SinoGenoMax Co., Ltd, China. The correct mutant plasmid was then transformed into *E. coli* BL21 (DE3) for expression.

## Additional file

Additional file 1: Figure S1. Multiple amino acid sequence alignment. Figure S2. SDS–PAGE analysis of recombinant TtAG at different stages of purification. (DOCX 985 kb)
