# Supplementary material for: Evaluation and directed evolution for thermostability improvement of a GH 13 thermostable α-glucosidase from Thermus thermophilus TC11
Source: BMC Biotechnol. 2015 Oct 21;15:97. doi: 10.1186/s12896-015-0197-x (PMC4618444; doi:10.1186/s12896-015-0197-x)
Supplement: Additional file 1: Figure S1. — Multiple amino acid sequence alignment. Figure S2. SDS–PAGE analysis of recombinant TtAG at different stages of purification. (DOCX 985 kb) [file 12896_2015_197_MOESM1_ESM.docx]

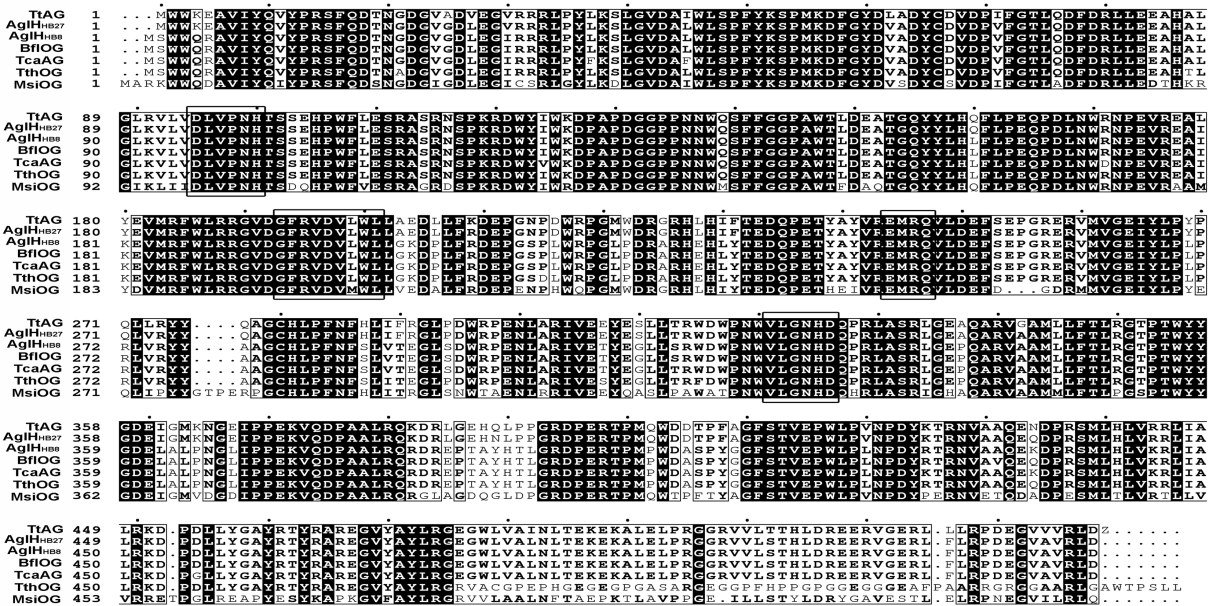


**Supplementary figure 1. Multiple amino acid sequence alignment.** The α-glucosidases from *Thermus thermophiles* TC11 (TtAG), *Thermus thermophilus* HB27 (AglH_HB27_, GenBank No. WP_011172564), *Thermus thermophilus* HB8 (AglH_HB8_, GenPept No. YP143747), *Bacillus flavocaldarius* (BflOG, GenBank No. BAB18518), *Thermus caldophilus* GK24 (TcaAG, GenBank No. AF096282), *Thermus* sp. RL (TthOG, GenBank No. EIA39407) and *Meiothermus silvanus* DSM 9946 (MsiOG, GenBank No.WP_013157279) were used. Identical residues are shaded black. The four short conserved regions of family GH 13 α-glucosidases are highlighted by a dotted black line.


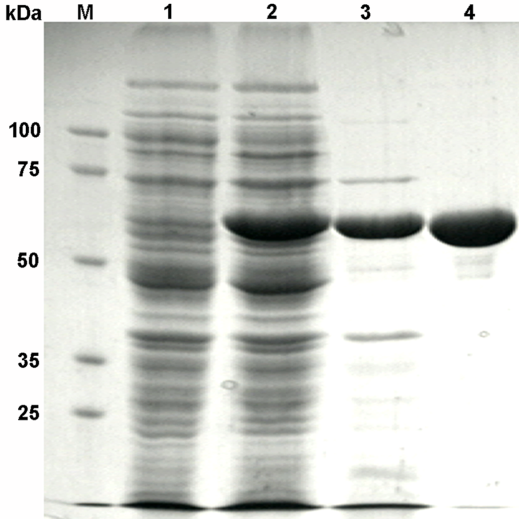


**Supplementary figure 2. SDS–PAGE analysis of recombinant TtAG at different stages of purification.** Lane 1, supernatant of crude extract from BL21(DE3) with pET28a; lane 2, supernatant of crude extract from BL21(DE3) with pET28a-*ttag*; lane 3, supernatant after heating treatment from BL21(DE3) with pET28a-*ttag*; lane 4, affinity (His-Tag column) chromatography-purified enzyme; lane M, molecular weight marker.
